# Effects of Manipulated Rainfall and Intraspecific Variation Within Dominant Species on Community Assembly: Insights From a Long‐Term Grassland Restoration Experiment

**DOI:** 10.1002/ece3.70571

**Published:** 2024-11-17

**Authors:** Zhe Ren, Sara G. Baer, Loretta C. Johnson, Matthew B. Galliart, David J. Gibson

**Affiliations:** ^1^ Department of Botany and Plant Pathology Purdue University West Lafayette Indiana USA; ^2^ School of Biological Sciences Southern Illinois University Carbondale Illinois USA; ^3^ Department of Ecology & Evolutionary Biology and Kansas Biological Survey & Center for Ecological Research University of Kansas Lawrence Kansas USA; ^4^ Division of Biology Kansas State University Manhattan Kansas USA; ^5^ Department of Biological Sciences Fort Hays State University Hays Kansas USA

**Keywords:** biological filter, community assembly, grassland, intraspecific trait variation, long term, multitrait space

## Abstract

Grasslands converted to agricultural land use can be reestablished by sowing seeds of native species and temporal dynamics of diversity under altered climate can inform community assembly in the context of global change. We quantified three aspects of diversity (species richness, phylogenetic diversity, and functional diversity) in restored prairie plots sown with different ecotypes of two dominant grass species and manipulated rainfall to understand the relative importance of abiotic filtering and population source of dominant species on community assembly. We also evaluated the contributions of intra‐ and interspecific variations in functional traits across plots sown with different ecotypes of dominant species. Since the fourth year of community establishment, species richness decreased over time as dominant species gradually established. Phylogenetic and functional diversity was unaffected by the ecotypic sources of dominant species during restoration. Experimental drought did not affect species richness, phylogenetic, or functional diversity. Community structure in the grasslands was mainly shaped by intraspecific, within‐population variation in the dominant species rather than by differences in traits among species. Our results showed that intraspecific biotic interactions contribute more than environmental filtering to community assembly in a tallgrass‐dominated prairie ecosystem.

## Graphical abstract

In restored prairie plots with different ecotypes of dominant grass species and manipulated rainfall, we examined the effects of abiotic filtering and population source on community assembly, finding that species richness declined over time as dominant species established, while phylogenetic and functional diversity remained unaffected. Our results indicated that intraspecific, within‐population variation in dominant species primarily shapes community structure, highlighting the greater role of biotic interactions over environmental filtering in prairie ecosystems.

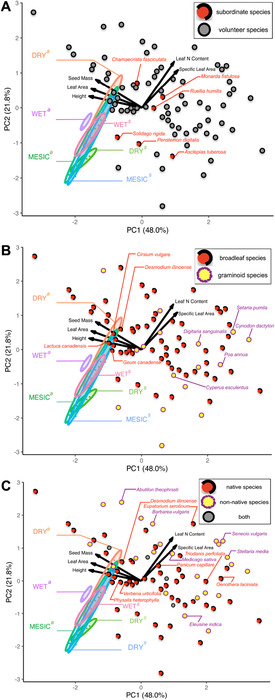

## 1 Introduction

Understanding factors influencing temporal dynamics of biodiversity during restoration is one of the key foci of ecological research (Pavoine and Bonsall 2011; Gibson et al. 2012; Baer, Gibson, and Johnson 2019). Traditional diversity indices measure community members as evolutionarily independent and ecologically equivalent but lack adequate details about how species are related and assembled in specific patterns (Arnan, Cerda, and Retana 2015). Therefore, additional diversity methods have been explored to provide important information about evolutionary history and trait patterns of communities: phylogenetic diversity measures the assembled evolution and history of species in a community, while functional diversity quantifies the states of morphological, physiological, and phenological traits affecting species' fitness (Webb et al. 2002; Petchey and Gaston 2006). Past efforts to substitute one diversity pattern with another have brought about criticism of the proxy diversity measures. For instance, using richness instead of trait‐based metrics oversimplifies diversity, ignores ecological redundancy, and misguides conservation efforts (Chave, Chust, and Thébaud 2007; Losos 2008). Kraft et al. (2007) found that local phylogenetic overdispersion reflects trait overdispersion only if traits are highly conserved. Kluge and Kessler (2011) observed no phylogenetic diversity pattern along elevation, despite varying in functional diversity. Spasojevic and Suding (2012) found no correlation between phylogenetic and functional diversity along resource–stress gradients, and E‐Vojtkó et al. (2023) noted that phylogenetic diversity rarely represents functional diversity in temperate vegetation.

Grassland biodiversity patterns vary across different scales and metrics such as species richness, evolutionary history, and functional traits of plant species (Khalil et al. 2018). Recent studies of grassland assembly have changed the emphasis from the straightforward measurement of species diversity to more process‐centered indicators, including assessing evolutionary‐ and trait‐based assembly drivers and determinants (Webb et al. 2002; Hardy and Senterre 2007; Pavoine, Baguette, and Bonsall 2010; Khalil et al. 2018; Jones, Barber, and Gibson 2019). Mechanistic studies on assembly drivers in tallgrass prairie, focusing on environmental factors like rainfall (Johnson et al. 2015; Knapp et al. 2024; Mount et al. 2024) and biotic drivers such as locally adapted seed sources of *Andropogon gerardi* (Galliart et al. 2020; Ren et al. 2023) and *Sorghastrum nutans* (Wilson et al. 2016; Vogel et al. 2018), can guide management decisions to avoid undesirable restoration outcomes (Baer, Gibson, and Johnson 2019; Jones, Barber, and Gibson 2019).

Knowledge of multidimensional diversity patterns of restored grasslands can serve as a critical tool in steering community assembly over time, thereby mitigating loss of biodiversity. In long‐term restoration efforts of North American grasslands, species losses have been observed (McLachlan and Knispel 2005; Twidwell et al. 2012; Young et al. 2015; McKone, Williams, and Beck 2021). However, functional and phylogenetic relationships in grasslands might not be necessarily connected to species richness (Belinchon, Hemrova, and Munzbergova 2019). Grassland functional and phylogenetic diversity could be expected to persist despite a decrease in species richness (Belinchon, Hemrova, and Munzbergova 2019). Historical climate changes could have driven greater evolutionary similarity within grassland communities (Li, Miller, and Harrison 2019; Harrison, Spasojevic, and Li 2020; Luong, Holl, and Loik 2021). Midolo, Kuss, and Wellstein (2021) further showed that increasing drought can reinforce trait similarities, such as seed mass and specific leaf area, linked to water availability in grasslands.

Recent trait‐based community studies on grassland restoration have highlighted the significant role of intraspecific trait variation (ITV). This variability is crucial for fostering species richness (Crawford et al. 2019) and maintaining diversity of functional traits (He et al. 2021). It also plays a key role in shaping competitive interactions (Fajardo and Siefert 2018; Carmona et al. 2019) and preserving genetic diversity (Zeldin et al. 2020). Additionally, ITV contributes to increasing adaptability (Lanuza et al. 2020) and elevating ecosystem stability (Lambert, Baer, and Gibson 2011). ITV can be influenced by phenotypic plasticity, environmental contexts, and evolutionary processes (Messier, McGill, and Lechowicz 2010; Violle et al. 2012). Plant species often show a high ITV in functional traits due to plasticity and heritable genetic variation (Violle et al. 2012; Siefert et al. 2015). ITV is considered a component of “internal filtering” to influence community assembly through biotic interactions, for example, competition or commensalism (Crawford et al. 2019). Yet, the exact mechanism by which ITV influences community assembly remains a subject of debate. On one hand, coexistence theory (Chesson 2000) states that ITV‐induced niche overlap exacerbates the dominance of the better competitor. In contrast, “individual variation” theories (Violle et al. 2012) declare that community assembly arising from ITV are challenging to model accurately while vital to the maintenance of diversity (Clark 2010).

While ITV is a major “internal filter,” climate stressors such as variation in rainfall are crucial “external filters” affecting grassland assembly (Funk 2021). Hallett et al. (2019) found that greater rainfall variability enhanced species coexistence in Californian grasslands. Manning and Baer (2018) noted that interannual rainfall variations influenced community assembly and ecosystem functioning in restored tallgrass prairie. Atkinson et al. (2023) highlighted that variation in rainfall during establishment significantly impacted trait diversity and biodiversity success in restored grasslands.

To better understand the relative importance of ITV as an internal filter and manipulated rainfall as an external filter on community assembly in restored grassland, we asked the following questions: (1) Does a manipulated rainfall × ITV interaction influence grassland diversity over time? (2) Does intraspecific trait variation influence grassland community assembly similarly to interspecific trait variation? We use a long‐term field experiment that contained plots established with different ecotypes of two dominant grass species and the same eight nondominant species with and without rainfall reduction treatments to test two hypotheses. First, we hypothesized that experimental drought would act as an environmental filter and decrease species richness while increasing evolutionary similarity and trait similarity compared to an ambient treatment. Previous studies showed that severe experimental drought can significantly decrease species diversity and exacerbate shifts in grassland community structure due to the local extinction of subordinate species (Tilman and Haddi, 1992; Smith et al. 2020; Knapp et al. 2024). Second, we hypothesized that intraspecific trait variation (ITV) serves as a more influential biotic filter than interspecific trait variations in shaping a restored grassland. For example, studies have indicated that ITV could have a predominant influence on grassland assembly, such as in mesic meadows (Volf et al. 2016), semiarid grasslands (Zhang et al. 2019), and urban–rural grassland gradients (Cochard et al. 2019).

## 2 Materials and Methods

### 2.1 Study Site and Seed Sources

A common garden consisting of plots sown with different ecotypes of dominant grass species was established in the spring of 2009 in Carbondale, Illinois (37°41′47.0″ N, 89°14′19.2″ W). This site receives an average annual rainfall of 1198 mm and the average mean temperature is 13.5°C (Galliart et al. 2020; NCEI, 2019). The site was under agricultural cultivation prior to common garden establishment and characterized as silt loam soils (Mendola et al. 2015).

Big bluestem (*A. gerardi*) and Indiangrass (*S. nutans*) were chosen as the dominant species for our tallgrass prairie restoration experiment as these C_4_ grasses dominate large areas of native tallgrass prairie (Risser et al. 1981). In the fall of 2008, seeds of *A. gerardi* and *S. nutans* were collected from three regions along a rainfall gradient from southern Illinois (WET; mean annual rainfall _[MAP]_ = 1097 mm) to eastern (MESIC; mean annual rainfall _[MAP]_ = 849 mm) and central (DRY; mean annual rainfall _[MAP]_ = 654 mm) Kansas (Johnson et al. 2015; Wilson et al. 2016). Genetic, phenotypic, and chemical variations confirmed the identity of *A. gerardi* ecotypes in the Great Plains (Gibson et al. 2013; Caudle et al. 2014; Gray et al. 2014; Johnson et al. 2015; Galliart et al. 2019, 2020). The mechanism behind the regional differences among *S. nutans* seed sources remained unknown, though ecotypes of *S. nutans* were anticipated to match the pattern of *A. gerardi* in the Great Plains (McMillan, 1959; Gustafson et al. 2014; Wilson et al. 2016).

Along with the two dominant species, seeds of eight subordinate species were also sown into the common garden plots (Table 1). The nondominant species sown included Canada wild rye (*Elymus canadensis*), butterfly milkweed (*Asclepias tuberosa*), partridge pea (*Chamaecrista fasciculata*), purple prairie clover (*Dalea purpurea*), wild bergamot (*Monarda fistulosa*), stiff goldenrod (*Solidago rigida*), foxglove beardtongue (*Penstemon digitalis*), and wild petunia (*Ruellia humilis*). The subordinate species were included to foster competitive dynamics akin to those found in prairie restorations (Johnson et al. 2015). Seeds of the eight subordinate species were purchased from a commercial provider (Ion Exchange, Inc.) volunteer species were allowed to establish in the plots, with no weeding done to preserve the natural species composition.

**TABLE 1:** Plant species and seeding density used for common garden establishment (Johnson et al. 2015).

Planted species		Family	Source	Seeding rate (seeds m^−2^)
Grasses	*Andropogon gerardi*	Poaceae	Local^a^	270
	*Sorghastrum nutans*	Poaceae	Local^b^	70
	*Elymus canadensis*	Poaceae	Commercial^c^	30
Forbs	*Asclepias tuberosa*	Apocynaceae	Commercial^c^	30
	*Chamaecrista fasciculata*	Fabaceae	Commercial^c^	30
	*Dalea purpurea*	Fabaceae	Commercial^c^, ^d^	30
	*Monarda fistulosa*	Lamiaceae	Commercial^c^	30
	*Solidago rigida*	Asteraceae	Commercial^c^	30
	*Penstemon digitalis*	Plantaginaceae	Commercial^c^	30
	*Ruellia humilis*	Acanthaceae	Commercial^c^	30
	Total seeds (m^−2^)			580

^a^ Seeds were collected from multiple remnants.

^b^ Seeds were collected from one remnant prairie within the native habitat for each ecotype (e.g., DRY ecotype in Hays, KS, USA, 38°51′13.2″ N, 99°19′08.6″ W; MESIC ecotype in Manhattan, KS, USA, 39°08′22.3″ N, 96°38′23.3″ W; and WET ecotype in Carbondale, IL, USA, 37°41′47.0″ N, 89°14′19.2″ W).

^c^ Seeds purchased from Ion Exchange Inc. Harpers Ferry, IA, USA.

^d^ *Dalea purpurea* was initially sown for the experiment but was absent in field surveys from 2012 to 2019.

### 2.2 Common Garden Establishment

The long‐term experiment contained a randomized complete block design. Four blocks contained three 4 × 4 m plots randomly assigned to be sown with one of three ecotypes (WET, MESIC, or DRY) of the dominant species (*A. gerardi* and *S. nutans*). The sown seed density of *A. gerardi* was 270 live seeds m^−2^ and *S. nutans* was at a density of 70 live seeds m^−2^ (live seed percentage was determined by the Kansas Seed Crop Improvement Center, Manhattan, Kansas, USA). Seeds of each subordinate species were added at a rate of 30 live seeds m^−2^ (Johnson et al. 2015). For a single plot, the total live seed density was 580 seeds m^−2^, as suggested for grassland restoration (Packard and Mutel 2005). The seed was mixed with damp sand, hand broadcast into plots, and raked into the soil (Johnson et al. 2015). The buffer zones between plots were seeded with little bluestem (*Schizachyrium scoparium*) and sideoats grama (*Bouteloua curtipendula*) supplied by a commercial seed company Ion Exchange Inc. (Johnson et al. 2015). Prescribed burning was applied in the site after the end of the growing season each year, starting from the fall of 2009 (Wilson et al. 2016).

In 2011, rainfall reduction shelters were installed according to a split‐plot design. Rainout shelters were placed in over half of each plot sown with a single ecotype of the dominant species. The shelters were designed to intercept 50% of ambient rainfall (Yahdjian and Sala 2002) and reduced rainfall by 34%–38%, based on measurements of rainfall and intercepted water collected in the site from June to September 2012 (Wilson et al. 2016). The size of shelter frames was 2.4 × 2.5 m, and each roof was constructed of clear acrylic, V‐shaped plates (0.13‐m wide and 2‐m long) spaced 20 cm apart. The roof was angled at a 20° slope to direct rain into a gutter on the low side, guiding it away from the plots (Yahdjian and Sala 2002). Shelters were placed in the field to cover a 2 × 2 m area of each plot. To minimize shading and warming greenhouse effects, we maintained a 150‐cm gap between the lower roof edge of the rainout shelters and the ground, preventing interference with the plant canopy (Kreyling et al. 2017). Kramer et al. (2018) found that the rainout shelters had little effect on the morphological traits of dominant species *A. gerardi* in the North American Tallgrass Prairie. All the experimental drought shelters were erected close to the beginning of the growing season, early June of each year, when a quarter of the mean annual cumulative temperature had elapsed (Johnson et al. 2015).

### 2.3 Plant Surveys

We identified and visually estimated percent cover of each species rooted in each of four 1 m^2^ (1 × 1 m) quadrats in each single plot (two quadrats in each of the ambient and reduced rainfall treatment). Plant surveys were conducted in late summer each survey year. Field surveys were conducted in 2012, 2014, 2018, and 2019.

### 2.4 Phylogeny and Functional Traits

The taxonomic name of each plant species was standardized with the Taxonomic Name Resolution System (TNRS) implemented in the R package “taxize” (version 0.9.98; Chamberlain and Szocs, 2013). We employed the largest fossil‐dated mega‐phylogeny for spermatophytes, GBOTB, comprising 79,881 taxa, as the basis to construct a phylogeny for plant species in our common garden site (Smith and Brown 2018). At the species level, 91 species from 32 families in the restoration experiment were identified in the latest mega‐phylogeny. Phylogeny in the site was performed using *phylo.maker* function in the “V.PhyloMaker” package version 0.1.0 (Jin and Qian 2019). We added sago palm (*Cycas revoluta*) as the outgroup. We employed scenario three and “build.nodes.1” in V.PhyloMaker. We eventually pruned the mega‐phylogeny to maintain only the plant species in the experiment.

We measured functional traits from 10 individuals for the three ecotypes of *A. gerardi* and *S. nutans* in late August 2019. We followed a standardized protocol to measure dominant species' traits (Cornelissen et al. 2003) of specific leaf area (cm g^−1^), height (cm), leaf nitrogen (N) content (mg g^−1^), leaf area (cm^2^), and seed mass (mg). These functional traits were selected to describe either interspecific or intraspecific competition relevant to nutrient and light uptake (Violle et al. 2012; Swenson et al. 2012; Lasky et al. 2014) and are considered advantageous compared to discrete traits since continuous traits can account for quantitative modeling and forecast plant functions (Swenson and Weiser 2010). We quantified specific leaf area (cm g^−1^) by dividing leaf fresh area by dry leaf mass. We acquired fresh leaf area (cm^2^) with an LI‐3000C leaf area meter (Licor, Lincoln, Nebraska, USA). We measured leaf dry mass (g) following oven drying at 45°C for 3 days (Khalil et al. 2018). We estimated leaf nitrogen content (mg g^−1^) with a Thermo Scientific Flash 2000 CNHSO Elemental Analyzer (Thermo Fisher Scientific, Waltham, Massachusetts, USA). We calculated the seed mass (mg) by weighing 1000 seeds. We measured functional traits of 39 subordinate and volunteer species collected from the same field in 2015 (Agronomy Research Center SIU, Carbondale, IL, USA; 37°41′47.0″ N, 89°14′19.2″ W). The traits were obtained from either 20 replicates (canopy % cover ≥ 10) or five replicates (canopy % cover < 10; Khalil et al. 2018). We utilized functional traits of the remaining 50 volunteer species available from the TRY plant trait database (version 5.0) if traits were unavailable at the time of surveys (Maitner et al. 2018; Kattge et al. 2020; Appendix S1). To ensure the quality of trait observations, we followed the standard data cleaning protocol for TRY database (Augustine et al. 2024) to retain continuous trait data that met the following criteria: (1) marked by TRY database as unduplicated (unique in the database), (2) represented as a mean or single observation (e.g., excluding minimum and maximum values), and (3) not marked as an outlier by TRY database (e.g., within three standard deviations of the species trait mean).

### 2.5 Data Analysis

We assessed species richness in each plot by averaging the abundance of individual species within each subplot. We estimated phylogenetic or functional mean pairwise distance by the standardized effect size (*sesmpd*) using the “picante” (version 1.8.2) R package (Kembel et al. 2010). Phylogenetic diversity (*PDsesmpd*) and functional diversity (*FDsesmpd*) are abundance‐weighted metrics calculated as:

$$\mathrm{PD}{\left(\text{or}\;\mathrm{FD}\right)}_{\text{sesmpd}}=\frac{{\text{Mean}}_{\mathrm{obs}}-{\text{Mean}}_{\mathrm{rand}}}{{\mathrm{SD}}_{\mathrm{rand}}},$$

where Mean_obs_ is the observed mean pairwise distance, Mean_rand_ is the mean of random mean pairwise distance, and SD_rand_ is the standard deviation of the random mean pairwise distance (Swenson 2014). Random communities were produced by random shuffling of taxa labels across the branching diagram's tips 999 times (Swenson 2014). Positive values of *sesmpd* suggest a high degree of trait or evolutionary dissimilarity, while negative values imply a low degree of trait or evolutionary dissimilarity. To ensure comparability across traits and mitigate biases from varying scales or units, we standardized functional traits to have a mean of zero and a standard deviation of one, and quantified *sesmpd* using the Gower distance (Swenson 2014). Values of *sesmpd* were quantified based on species abundance in plots, that is, they were abundance weighted by using relative percentage cover of each species (Webb et al. 2002; Kembel et al. 2010). We used a repeated measures generalized linear mixed model (GLMM) with a Poisson distribution and a log link function to analyze the discrete response variable of species richness (number of species). Experimental drought, ecotype, year, and their interactions were included in the model as the fixed factors. We utilized a repeated measures linear mixed model (LMM) to examine the effects of experimental drought, ecotype, year, and their interactions on the continuous response variables *PDsesmpd* or *FDsesmpd*. Block and plot (as repeated measures) were treated as random factors in GLMM and LMM. For post hoc evaluation, we applied Tukey's multiple comparison test. We used Cohen's *d* to estimate effect size to show the magnitude of temporal change in *PDsesmpd* or *FDsesmpd*. An effect size of 0.7 means the mean response of 1 year is 0.7 standard deviations different from another year. Temporal differences are considered trivial (0 < *d* ≤ 0.2), small (0.2 < *d* ≤ 0.5), moderate (0.5 < *d* ≤ 0.8), and strong (*d* > 0.8; Cohen 1992). We used the R (version 4.0.2) packages, including “lme4” (version 1.1.23), “multcomp” (version 1.4.13), and “emmeans” (version 1.5.0) for the models (Hothorn, Bretz, and Westfall 2008; Bates et al. 2015; Lenth 2016; R Core Team 2020; Appendix S2).

To evaluate the effect of internal (ITV) and external (rainfall) filters in determining grassland community assembly, we assessed trait statistics (*T*‐statistics) to estimate where functional traits were most significant with different ecotypes of the dominant species (Violle et al. 2012). Functional traits of a community were represented from each ecotype (WET, MESIC, or DRY) of the dominant species *A. gerardi* (Gray et al. 2014; Galliart et al. 2020) and *S. nutans* (Khalil, Gibson, and Baer 2019). Three components of *T*‐statistics were summarized to partition phenotypic variance in traits into three organizational levels: (i) $T_{\text{internal}}$ is the ratio of trait variance within ecotype (e.g., ITV within a WET ecotype) to total trait variance within a plot (e.g., trait variation of all individuals within a plot sown with the WET ecotype). The $T_{\text{internal}}$ component serves as a measure of internal filtering, aiming to assess the role of ITV in shaping community assembly, highlighting that the two individuals are members from the same population and can show more similar trait values than two individuals selected randomly from a plot (Taudiere and Violle 2016). (ii) $T_{\text{external}\;\alpha }$ is the ratio of trait variance within a plot (e.g., WET‐ecotype plot) to trait variance of the whole common garden experiment (e.g., trait variance of all individuals across plots sown with WET, MESIC, and DRY ecotypes of dominant species). Thus, $T_{\text{external}\;\alpha }$ can be interpreted as a measure of external filtering (e.g., controlled by manipulated rainfall) while accounting for trait variation of individuals (Jordani et al. 2019). (iii) $T_{\text{external}\;\beta }$ is the ratio of trait variance within a plot (e.g., WET‐ecotype plot) relative to the total trait variance in the common garden experiment as a quantity of the power of external filtering without taking intraspecific variation into account. ITV among three ecotypes were summarized in a principal component analysis (PCA). Ellipses with a 68% probability (i.e., the proportion of samples within one standard deviation) were added around points from each ecotype for both dominant grasses to visualize the degree of intraspecific trait variability (Vu 2011). We computed PCA with the *prcomp* function in “ggbiplot” (version 0.55) package in R software (version 4.0.2; R Core Team 2020).

We utilized standardized effect sizes (*SES*) of *T*‐statistic values to test the deviation of observed trait distributions from randomization (*n* = 999 permutations). SES was calculated:

$$\mathrm{SES}=\frac{I_{\mathrm{obv}}-I_{\mathrm{sim}}}{S_{\mathrm{sim}}},$$

where $I_{\mathrm{sim}}$ and $S_{\mathrm{sim}}$ are respectively the mean and the standard deviation of the randomized values of trait and $I_{\mathrm{obv}}$ is the observed value of trait. SES estimates the number of standard deviations which differentiate the observed trait values from the average values of the simulated communities (Gotelli and McCabe 2002). A negative SES value indicates the *T*‐statistic value lower than random expectation, representing the overlap of trait distribution less than expected value by chance (Jordani et al. 2019). By contrast, a positive SES value suggests the *T*‐statistic value higher than random expectation, showing trait distribution overlapped more than null expectation. The trait analysis was performed in R version 4.0.2 (R Core Team 2020; Appendix S2), using *tstats* function in the “cati” (version 0.99.4) package for the *T*‐statistics (Taudiere and Violle 2016).

## 3 Results

### 3.1 Impact of Rainfall × ITV on Grassland Diversity

**FIGURE 1:**
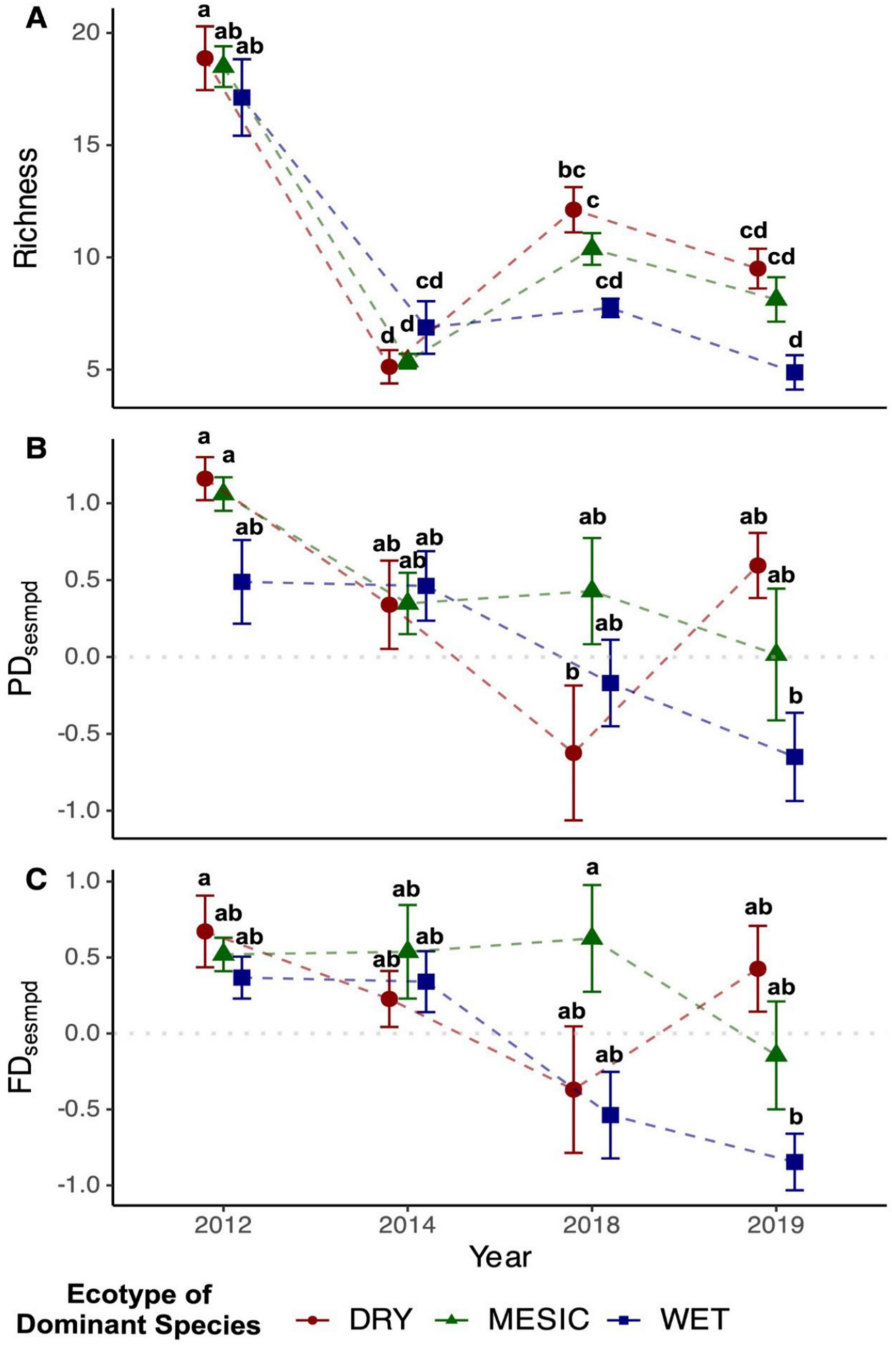
Results showing the interactive effect of dominant grass ecotype (DRY, MESIC, or WET) and year (2012, 2014, 2018, or 2019) on (A) species richness (number of species m^−2^), (B) phylogenetic diversity (*PDsesmpd*), and (C) functional diversity (*FDsesmpd*). Values of metrics from the same year are horizontally jittered to aid visualization. Above data points and error bars sharing the same letter indicate nonsignificant differences (*p* > 0.05).

To address the question, “(1) Does a manipulated rainfall × ITV interact to influence grassland diversity overtime?,” we surveyed 91 species comprising 32 plant families. The most abundant species were among five angiosperm families including Asteraceae (*n* = 21 species), Poaceae (*n* = 19 species), Fabaceae (*n* = 8 species), Brassicaceae (*n* = 4 species), and Convolvulaceae (*n* = 3 species). Species richness showed no response to the experimental drought (χ^2^ < 0.01, df = 1, *p* = 0.98), or interactions with drought (ecotype × experimental drought: χ^2^ = 2.07, df = 2, *p* = 0.36; year × experimental drought: χ^2^ = 0.58, df = 3, *p* = 0.90). There was an ecotype × year interaction on richness (*χ* ^2^ = 15.44, df = 6, *p* = 0.02; Figure 1a). Species richness in local WET ecotype plots declined during the first two surveyed years from 2012 to 2014, though there were no differences in the WET‐ecotype plots in the following years (Figure 1a: blue line). Species richness in nonlocal DRY‐ecotype plots in 2012 was higher than all the plots in the later years (Figure 1a).

Overall phylogenetic diversity showed a structural shift of species from a distant evolutionary relationship in 2012 (*PDsesmpd* = 0.90 ± 0.12) to a random evolutionary relationship in 2019 (*PDsesmpd* = −0.01 ± 0.21), accompanied by a Cohen's *d* effect size of 1.13, indicating a substantial decline in *PDsesmpd* values from early year 2012 to later year 2019. Phylogenetic diversity showed no response to experimental drought (*F*_1,63_ = 0.61, *p* = 0.44), ecotype × experimental drought (*F*_2,63_ = 0.20, *p* = 0.82), or year × experimental drought (*F*_3,63_ = 0.24, *p* = 0.87) interactions. There was an ecotype × year effect (*F*_6,63_ = 2.43, *p* = 0.04) on phylogenetic diversity (*PDsesmpd*; Figure 1b) resulting from differences between 2012 and 2019. Specifically, species in local WET‐ecotype plots in 2019 were more closely related evolutionarily than species in nonlocal MESIC‐ or DRY‐ecotype plots in 2012 (Figure 1b). Species in nonlocal DRY‐ecotype plots in 2018 were also more closely related evolutionarily than species in non‐local MESIC‐ or DRY‐ecotype plots in 2012. In contrast, no ecotype effect occurred between 2014 and 2018.

Overall functional diversity also showed a trait compositional shift for co‐occurring species from high dissimilarity in early year 2012 (*FDsesmpd* = 0.52 ± 0.10) to a random relationship of trait pattern in later year 2019 (*FDsesmpd* = −0.18 ± 0.19), accompanied by a Cohen's *d* effect size of 0.92, indicating a substantial decrease in *FDsesmpd* values from 2012 to 2019. Besides, functional diversity showed no response to experimental drought alone (*F*_1,63_ = 1.22, *p* = 0.27), or ecotype × experimental drought (*F*_2,63_ = 0.01, *p* = 0.988), or year × experimental drought (*F*_3,63_ = 0.05, *p* = 0.98) interactions. There was an ecotype × year effect (*F*_6,63_ = 2.33, *p* = 0.04) on functional diversity (*FDsesmpd*; Figure 1c). Species in local WET‐ecotype plots in 2019 showed higher trait similarity than species in nonlocal MESIC‐ecotype plots in 2018 and DRY‐ecotype plots in 2012 (Figure 1c).

### 3.2 Intraspecific Versus Interspecific Trait Variation in Grassland Assembly

To address the question, “(2) Does intraspecific trait variation influence grassland community assembly similarly to interspecific trait variation?,” we first summarized the trait values (mean ± standard error) for the three ecotypes of the dominant species (both *A. gerardi* and *S. nutans*; Table 2) to assess ITV among the dominant grasses. In local WET‐ecotype plots, *A. gerardi* exhibited heights 32% and 42% greater than those in nonlocal MESIC‐ or DRY‐ecotype plots, respectively, while *S. nutans* displayed heights 24% and 23% higher in local WET‐ecotype plots compared to nonlocal MESIC‐ or DRY‐ecotype plots. The leaf area of *A. gerardi* in WET‐ or DRY‐ecotype plots increased by 33% and 28%, respectively, compared to MESIC‐ecotype plots, with no difference observed among *S. nutans* ecotypes. In local WET‐ecotype plots, *A. gerardi* exhibited 15% and 11% higher seed mass compared to nonlocal MESIC‐ or DRY‐ecotype plots, while in MESIC‐ecotype plots, *S. nutans* displayed 20% and 26% greater seed mass compared to WET‐ or DRY‐ecotype plots, respectively. The specific leaf area of both species did not differ across ecotypes. Finally, in WET‐ or MESIC‐ecotype plots, *S. nutans* leaf nitrogen content was 58% and 44% higher than in DRY‐ecotype plots, respectively, with no difference observed among *A. gerardi* ecotypes.

**TABLE 2:** Trait measurements (mean ± standard error) of ecotypes (WET, MESIC, or DRY) of each dominant grass species ( *Andropogon gerardi* or *Sorghastrum nutans* ).

Functional trait	*Andropogon gerardi* (*n* = 10 per ecotype)			*Sorghastrum nutans* (*n* = 10 per ecotype)		
	WET	MESIC	DRY	WET	MESIC	DRY
Height (cm)	286.7 ± 2.8 a	217.4 ± 3.23 b	201.3 ± 4.47 c	257.0 ± 7.23 a	206.9 ± 5.57 b	208.5 ± 4.02 b
Leaf area (cm^2^)	37.1 ± 1.28 a	27.9 ± 1.38 b	35.7 ± 2.49 a	30.3 ± 2.17 a	28.8 ± 2.24 a	28.8 ± 2.27 a
Seed mass (mg)	3.1 ± 0.06 a	2.7 ± 0.11 b	2.8 ± 0.06 b	2.0 ± 0.05 b	2.4 ± 0.04 a	1.9 ± 0.03 b
Specific leaf area (cm^2^ g^−1^)	170.0 ± 6.62 a	149.2 ± 20.43 a	210.1 ± 21.95 a	142.7 ± 12.61 a	149.2 ± 21.13 a	137.6 ± 6.92 a
Leaf N content (mg g^−1^)	11.2 ± 0.56 a	10.6 ± 0.78 a	11.7 ± 0.78 a	11.4 ± 0.54 a	10.4 ± 0.56 a	7.2 ± 0.53 b

*Note:* Sample sizes (*n*) refer to the number of individual plants from which traits were measured. (mean ± standard error) followed by identical letters were not significantly different from each other (experiment‐wide *α* = 0.05, Tukey adjusted).

**FIGURE 2:**
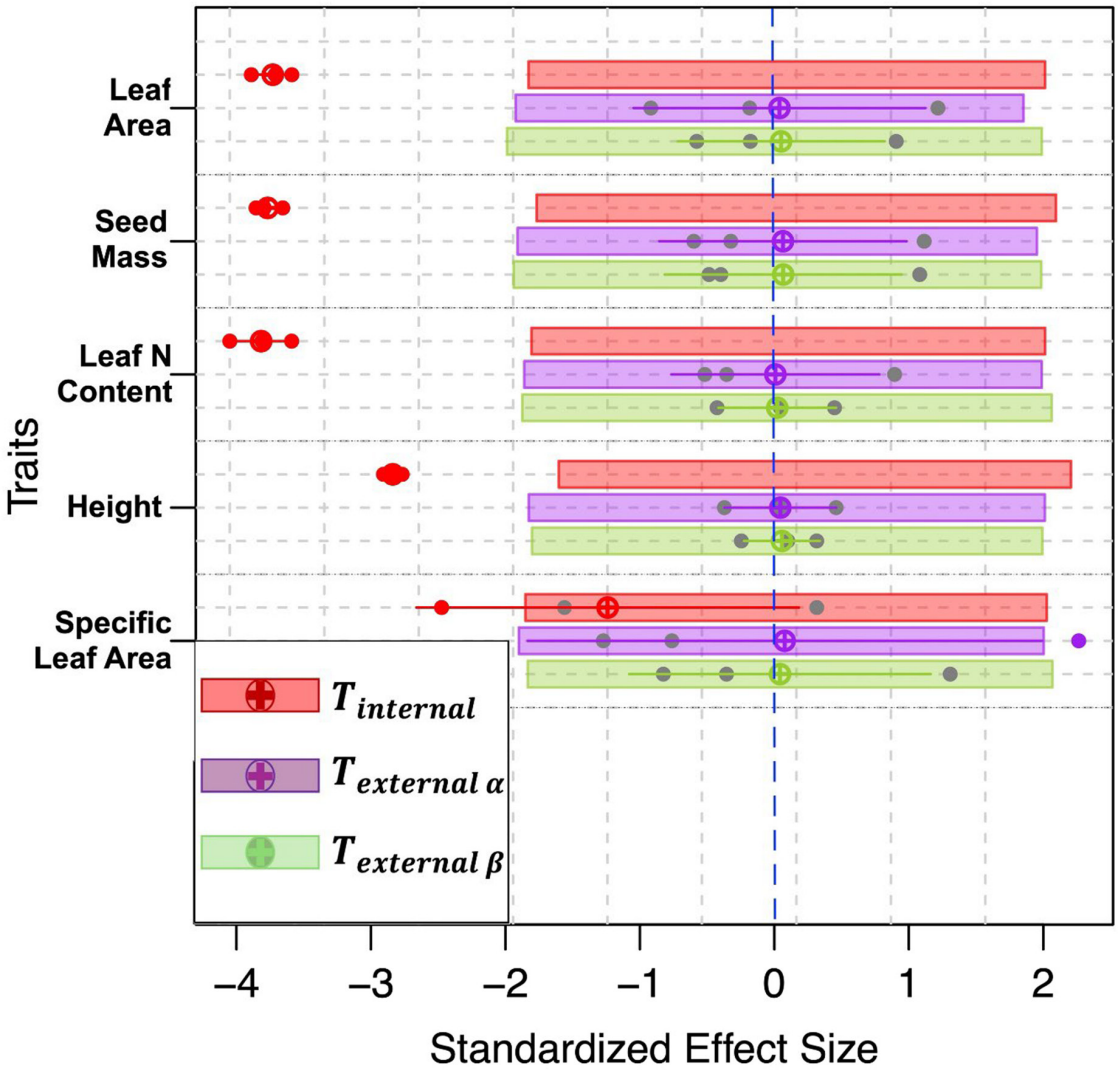
Standardized effect size (SES) of *T*‐statistics for the five traits: leaf area (cm^2^), seed mass (mg), leaf N content (mg g^−1^), height (cm), and specific leaf area (cm^2^ g^−1^) collected from the common garden experiment. The horizontal axis (SES) was employed to quantify the magnitude of changes, enabling comparison across distinct trait measures. Colored dots represent the SES value for plots planted with one dominant species ecotype (e.g., DRY, MESIC, or WET) when different from the null model. T_internal_ = the ratio of trait variance within ecotype (e.g., intraspecific variation of WET ecotype for dominant species) relative to total trait variance within the plot (e.g., including both intraspecific and interspecific variations); *T*_external α_ = the ratio of trait variance within a plot relative to trait variance of all plots in the common garden experiment; and *T*_external β_ = the ratio of trait variance within a plot relative to trait variance of all plots in the common garden experiment, excluding intraspecific trait variation. The crossed circles and the segments represent the mean and standard deviation of the SES values for a given *T*‐statistic and a given trait. For a given T‐statistic, the mean SES (crossed circle) is significantly different from the null distribution if not embedded within the colored bar (e.g., $T_{\text{internal}}$). The more the SES value departs from the null model, the stronger the filtering effect.

**FIGURE 3:**
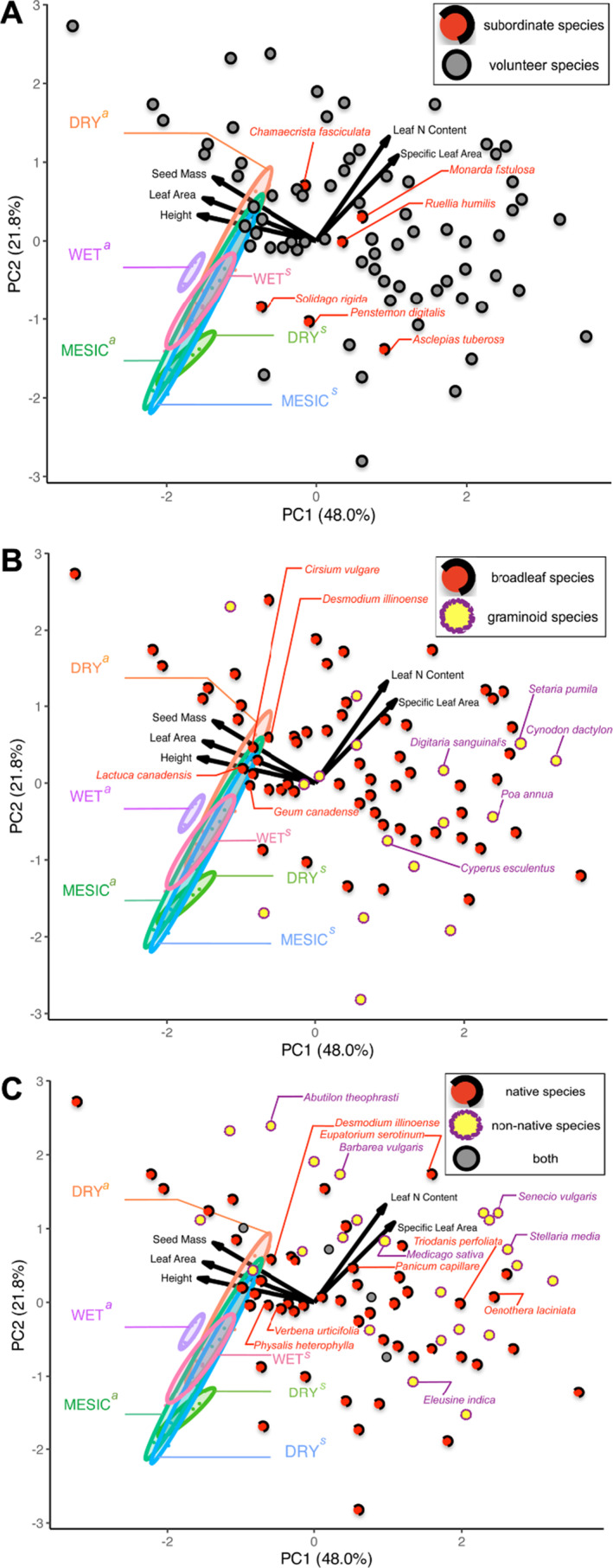
Principal components analysis (PCA) summarizing ITVs among ecotypes (DRY, MESIC, or WET) of dominant species (*a* = *Andropogon gerardi* ; *s* = *Sorghastrum nutans* ) in multivariate trait space. The ellipses are 68% data ellipses for *A. gerardi* and *S. nutans* . Dots represent the positions of nondominant species within PCA trait space grouped by (A) their roles in communities: red dots with dashed circles = subordinate species sown with *A. gerardi* and *S. nutans* in 2008 (Table 1); gray dots with solid circles = volunteer species, (B) their morphological features: red dots with dashed circles = broadleaf species; yellow dots with rough circles = graminoid species, and (C) their nativeness: red dots with dashed circles = native species; yellow dots with rough circles = non‐native species; and gray dots with solid circles = species can be both native and non‐native. Information on whether a species is native or non‐native to Illinois, USA was obtained from the US Department of Agriculture (USDA) PLANTS Database (https://plants.usda.gov). Representative species are labeled with their scientific names: subordinate (red) in A, broadleaf (red) or graminoid (purple) in B, and native (red) or non‐native (purple) in C. The solid arrowed lines show the direction and loadings of the traits including height, leaf area, seed mass, leaf N content, and specific leaf area.

To evaluate the effect of internal and external filters in determining community assembly, we assessed the departure of observed estimates of *T*‐statistics from randomized values for five functional traits (Figure 2), including leaf area, seed mass, leaf N content, height, and specific leaf area. The result was mostly consistent across different traits. $T_{\text{internal}}$values were calculated to measure internal filtering in grassland assembly. The mean estimates of $T_{\text{internal}}$ were significantly less than expected by chance for all the traits, except specific leaf area, which was the only trait showing mean $T_{\text{internal}}$ value within null expectation (Figure 3: red markings). Hence, within each community (planted with one of the three dominant species ecotypes WET, MESIC, or DRY), there was minimal overlap among species in terms of trait distributions when considering intraspecific variations. In other words, two individuals from a dominant species ecotype exhibited more similar trait values than two individuals selected randomly from the same community. In contrast, the $T_{\text{external}\;\alpha }$ values used for measuring external filtering did not deviate from null expectations on average for most communities (Figure 2: purple markings). Thus, two individuals randomly selected from a community planted with a particular ecotype were not necessarily more comparable or more distinctive than two individuals randomly selected from the entire common garden experiment (Jordani et al. 2019). In the same way, $T_{\text{external}\;\beta }$ values quantified the overlap among community trait distributions within the whole common garden without considering ITVs. The lack of departure for $T_{\text{external}\;\beta }$ values from null expectations showed that there was limited overlap in trait distributions between communities when focusing on population‐level trait estimates (Jordani et al. 2019, Figure 2: green markings).

Overall, the PCA (Figure 3a) revealed that the ellipse of local *A. gerardi* WET‐ecotype was much smaller compared with the ellipses of nonlocal MESIC‐ or DRY‐ecotype of *A. gerardi*. In contrast, the ellipses among ecotypes of *S. nutans* mostly overlapped (e.g., MESIC and DRY, MESIC and WET). Our result showed that the multidimensional trait overlaps of dominant species was shaped by interspecific (e.g., between *A. gerardi* and *S. nutans*) and ITVs (e.g., among ecotypes) of functional traits. Many broadleaf species, including common thistle (*Cirsium vulgare*), Illinois ticktrefoil (*Desmodium illinoense*), white avens (*Geum canadense*), and tall lettuce (*Lactuca canadensis*), exhibited a multitrait space close to dominant *A. gerardi* or *S. nutans* in the PCA (Figure 3b; nondominant species represented as colored dots instead of name/code labels due to significant label overlap in multitrait PCA space). In contrast, graminoids such as Bermuda grass (*Cynodon dactylon*), yellow nutsedge (*Cyperus esculentus*), large crabgrass (*Digitaria sanguinalis*), annual bluegrass (*Poa annua*), and yellow foxtail (*Setaria pumila*) occupied a multitrait space opposite to the two dominant species (Figure 3b). Native broadleaf species (Figure 3c) such as late boneset (*Eupatorium serotinum*), cutleaf evening primrose (*Oenothera laciniata*), and clasping bellflower (*Triodanis perfoliata*) also occupied a trait space opposite to the two dominant species in the PCA. Other natives such as Illinois ticktrefoil (*D. illinoense*), clammy groundcherry (*Physalis heterophylla*), and white vervain (*Verbena urticifolia*) were close to the dominant species. Most non‐native species, particularly agricultural weeds such as velvetleaf (*Abutilon theophrasti*), goosegrass (*Eleusine indica*), groundsel (*Senecio vulgaris*), and chickweed (*Stellaria media*), exhibited a multitrait space distinct from the dominant species (Figure 3c). Furthermore, height, leaf area, and seed mass showed high negative loadings on PC_1_ axis, while leaf nitrogen content and specific leaf area showed relatively high positive loadings on both axes.

## 4 Discussion

Manipulated rainfall did not affect plant diversity in our grassland community common garden experiment, indicating that developing tallgrass prairie may be resilient to less precipitation in the initial decade of restoration. Our first hypothesis that drought lowers species richness and increases evolutionary and trait similarity was not supported because there were no observed effects of the experimental drought treatment or its interaction with ecotype or year on species richness, phylogenetic (*PD‐*), and functional diversity (*FD‐sesmpd*). Yue et al. (2020) also reported a similar result following a meta‐analysis of experimental rainfall manipulations, discovering no overall treatment effect on plant diversity on all levels. Moreover, Komatsu et al. (2019) synthesized studies on manipulating precipitation either experimentally increased or reduced and found no effect of drought on taxonomic diversity. Our study site in southern Illinois, USA is located on the mesic edge of North American Tallgrass Prairie. Korell et al. (2021) studied 74 rainfall control experiments and found those plant communities in relatively wetter regions are often less sensitive to predicted variations in rainfall than water‐limited ecosystems. Although experimental drought might be expected to function as an abiotic filter by decreasing the possibility that certain plant species with lower drought tolerance will establish, there can be no correlation between grassland phylogenetic diversity and manipulated rainfall (Barber et al. 2017). This result supports a previous study that grassland responses to rainfall were not phylogenetically conserved (Bennett and Cahill 2013; Luong, Holl, and Loik 2021). Moreover, our result showed no effect of drought on plant functional diversity. It reinforced that trends in grassland functional diversity were not necessarily linked to loss of species during restoration, and variability of functional diversity is less prone to be only shaped by experimental treatments (Miller et al. 2019; Zuo et al. 2021; Karimi et al. 2022). Although our findings indicated no impact from the rainout shelter roofs, we cannot completely rule out the possibility of unwanted side effects on the microenvironment.

Species richness decreased significantly during the early years of assembly, coinciding with the increasing dominance of sown grasses (Ren et al. 2023). Similar declines in richness have been observed during the initial years of restoration in other grasslands. For example, the increased density of dominant species could lead to richness losses (Keddy et al. 2006; Avolio et al. 2019). Declines in richness may also result from decreased financial and labor resources postplanting, as long‐term maintenances are essential for high species diversity (Luong, Holl, and Loik 2021; Luong, Press, and Holl 2023). Contrary to the trend of decreasing richness, ecotypic effects on phylogenetic and functional diversity were generally minimal. However, our findings suggest that communities with locally adapted WET ecotypes, benefiting from a home‐site advantage, displayed a trajectory of increasing evolutionary and trait similarity over time, contrasting with communities hosting nonlocal ecotypes (Johnson et al. 2015; Mendola et al. 2015; Wilson et al. 2016). Previous studies showed that genetic differences shaped the competitive traits of the dominant grasses, causing *A. gerardi* from wet regions to display greater canopy cover, leaf count, stem diameter, and maximum leaf width compared to those from xeric areas (Kramer et al. 2018; Galliart et al. 2020).

Despite rejecting our first hypothesis regarding experimental drought's influence on biodiversity, we observed ecotype × year effect on functional (*FD‐*) and phylogenetic diversity (*PD‐sesmpd*) in the experiment. Previous studies showed that changes in the ITV of dominant grasses could alter grassland functional or phylogenetic diversity by impacting nondominant species (Gustafson et al. 2014; Khalil, Gibson, and Baer 2019). For example, cultivars impacted grassland phylogenetic diversity more than noncultivar population sources of a dominant grass species (Khalil, Gibson, and Baer 2017) by reducing the abundance of an evolutionarily distinct community of less closely related subordinate species. Khalil, Gibson, and Baer (2017) showed diversity patterns varied among metrics: phylogenetic and functional diversity were maintained at constant levels while taxonomic diversity declined during restoration. Purschke et al. (2013) also found contrasting changes in taxonomic, functional, and phylogenetic diversity within a chronosequence during a long‐term seminatural grassland succession. Understanding the role of intraspecific variation during grassland restoration is essential to inform predictions of how temperate grassland ecosystems will respond to global climate change (Baer, Gibson, and Johnson 2019). Furthermore, understanding the relative importance of evolutionary history and environmental conditions on the dominant grasses is necessary to determine the best sources of seed materials for prairie restoration and forecast ecosystem response to biotic or environmental assembly drivers (Mendola et al. 2015).

Our trait‐based analysis indicated variability in the grassland communities was principally due to within‐population trait variation resulting from ecotypic differentiation in the dominant species rather than differences between species in a community. This result supports our second hypothesis that an internal biotic filter plays a key role in grassland assembly with a less important external environmental filter. The ITV of dominant species as a primary internal filter significantly influences restoration efforts, ecosystem functions, environmental filtering, and species coexistence (Laughlin et al. 2012; Hart, Schreiber, and Levine 2016). Overall, the global pattern of plant traits showed that ITV constituted 32% of trait variation between communities and 25% within communities (Siefert et al. 2015). Although patterns of trait variation within species might seem idiosyncratic, the inter‐ and intraspecific variations of functional traits can be interpreted by environmental context, functional tactics, and evolutionary history (Sandel et al. 2021). Nevertheless, a limitation of trait‐based analyses is its reliance on empirical correlations and null models (Swenson 2014). Consequently, our analyses on functional traits did not encompass all life stages of dominant species and their interactions with all possible volunteer species, which could connect grassland function and phylogeny to the demography of dominant species (Enquist et al. 2015).

We found little support for an influence of an external filter on the communities planted with different dominant grass ecotypes. Similarly, Khalil, Gibson, and Baer (2019) showed that ITV as an internal filter was predominant among functional traits rather than trait variation among species as the external filter in restored grassland in southern Illinois. Fang et al. (2019) found that ITV analyses showed the importance of limiting similarity in driving community assembly at an early stage of succession. We also observed a strong internal filter effect on most functional traits, including height, seed mass, leaf area, and leaf N content in grassland communities, implying the internal filter with a low overlap in trait distributions. This less‐than‐random (negative SES value) trait overlap indicated that ITV among dominant species in our study, was largely driving the restored grassland assembly process (Khalil, Gibson, and Baer 2019). Likewise, Crawford et al. (2019) found that intraspecific variation played pivotal roles in grassland assembly processes. The PCA result revealed that the local WET‐ecotype of dominant species had a lower ITV compared to nonlocal ecotypes. Non‐native volunteer species such as rocketcress (*Barbarea vulgaris*), alfalfa (*Medicago sativa*), and native volunteer species such as witchgrass (*Panicum capillare*), which were exclusive to local WET‐ecotype plots, displayed traits such as higher leaf nitrogen content and specific leaf area than the dominant species *A. gerardi*. The differences in traits between dominant and nondominant species suggest that many volunteer species might occupy niches that are different from those of the dominant species due to limiting similarity.

In general, our results highlight the importance of integrating interspecific and intraspecific trait variabilities. We focused on functional traits to comprehend better how trait variability is coupled with species coexistence (Jordani et al. 2019). Future empirical and experimental studies should investigate ongoing theoretical research on ITV, such as the eco‐evolutionary theory of community structure (Wickman, Koffel, and Klausmeier 2023) and the niche packing hypothesis (Violle et al. 2012). These investigations are necessary to examine the distinctive origins of variability in plant traits and how they contribute to community assembly in restored grasslands. Embracing diverse practice and management strategies is crucial for enhancing ecological restoration efforts. These strategies may include selecting locally adapted seed sources or vegetative propagules to promote survival and growth, conducting long‐term monitoring to anticipate future challenges, and identifying native populations that thrive under controlled conditions, such as manipulated rainfall, to foster establishment in naturally variable environments.

## Author Contributions

**Zhe Ren:** conceptualization (lead), data curation (equal), formal analysis (lead), investigation (equal), methodology (equal), project administration (lead), resources (equal), software (lead), visualization (lead), writing – original draft (lead), writing – review and editing (equal). **Sara G. Baer:** investigation (equal), project administration (equal), resources (equal), writing – review and editing (equal). **Loretta C. Johnson:** funding acquisition (equal), project administration (equal), resources (equal), writing – review and editing (equal). **Matthew B. Galliart:** methodology (equal), project administration (equal), resources (equal), writing – review and editing (equal). **David J. Gibson:** data curation (equal), funding acquisition (equal), investigation (equal), methodology (equal), project administration (supporting), resources (equal), supervision (supporting), writing – review and editing (equal).

## Conflicts of Interest

The authors declare no conflicts of interest.

### Open Research Badges

This article has earned an Open Data badge for making publicly available the digitally‐shareable data necessary to reproduce the reported results. The data is available at (insert provided URL from Open Research Disclosure Form).

## Supporting information


**Appendix S1.** Supporting Information.

**Appendix S2.** Supporting Information.

## Data Availability

The data and codes used in this study are available with the corresponding Supporting Information.
